# Solvent-Controlled Strategy for Color-Tunable Fluorescence Carbon Dots and Their Application in Light-Emitting Diodes

**DOI:** 10.3390/molecules29194552

**Published:** 2024-09-25

**Authors:** Yuhua Zhang, Hong Zhao

**Affiliations:** 1 School of Pharmacy, Shandong Second Medical University, Weifang 261053, China; zhangyh@sdsmu.edu.cn; 2 School of Chemical Sciences, University of Chinese Academy of Sciences, Beijing 100049, China

**Keywords:** solvent-controlled strategy, multicolor carbon dots, sp^2^-conjugated domains, fluorescence mechanism, light-emitting diodes

## Abstract

Carbon dots (CDs) offer tremendous advantages in the fields such as bioimaging, sensing, biomedicine, catalysis, information encryption, and optoelectronics. However, the inherent challenge is synthesizing CDs with a full-spectrum emission, as most CDs typically produce only blue or green emissions, which severely hinder further investigation into their fluorescence mechanism and restrict their broader applications in light-emitting diodes (LEDs). In this work, we reported a solvent-controlled strategy for the preparation of multicolor CDs with blue, yellow, and red emissions, using *o*-phenylenediamine (*o*PD) and ionic liquid 1-butyl-3-methylimidazolium hexafluorophosphate (BmimPF_6_) as precursors. The detailed characterizations proved that a solvent with a lower boiling point and lower solubility of precursors resulted in a higher degree of dehydration and carbonization process, thereby increasing carbon cores with sp^2^-conjugated domains and nitrogen doping and further reducing the bandgap energies, causing a significant redshift emission from blue to red. The underlying fluorescence mechanism of the prepared multicolor CDs was contributed to the surface state. Eventually, blue-, yellow-, and red-emitting CDs based on poly(vinyl alcohol) (PVA) films and colorful LEDs devices were fabricated by dispersing the as-synthesized CDs into a PVA solution. The proposed solvent-controlled strategy for multicolor CDs preparation will be helpful for fully utilizing the advantages of CDs and expanding their applications.

## 1. Introduction

Carbon dots (CDs), as an emerging type of zero-dimensional carbon-based fluorescent nanomaterials, have gained huge interest due to their fascinating advantage in tunable fluorescent emission, chemical inertness, excellent biocompatibility, high water solubility, high quantum yields (QYs), cost-effective preparation, and low toxicity. Since being reported in 2004 [1], CDs have a significant potential for application in the fields of sensors [2,3,4], bioimaging [5], drug delivery [6], cancer diagnosis [7,8], catalysis [9,10], information encryption [11,12], optoelectronic devices [13,14], and so on. In the early studies, researchers focused on developing various preparation methods and carbon sources. Unfortunately, the majority of the prepared CDs are known to emit predominantly strong blue or green fluorescence, seriously limiting their applications in many fields. Furthermore, the diversity of precursors and synthetic methods made the prepared CDs with complex compositions and structures, which brings certain challenges to study the fluorescence mechanism. To date, the underlying fluorescence mechanism of CDs is debatable and even contradictory. A comprehensive understanding of the fluorescence mechanism of CDs is essential, not only for elucidating their structural chemistry, but also for guiding their large-scale synthesis. Therefore, synthesizing CDs with full-spectrum emission can significantly broaden their application scope, enhancing capabilities in deep-tissue imaging and full-color LEDs. Additionally, it facilitates a deeper understanding the fluorescence mechanisms of CDs.

In recent years, many remarkable efforts have been made to synthesize CDs with full-color emission though various methods, aiming to elucidate the origins of their emission properties [15,16,17,18,19,20,21,22]. For instance, Jiang et al. successfully synthesized blue-, green-, and red-emitting CDs using three different phenylenediamine isomers though a facile solvothermal method [15]. They discovered that the variations in fluorescence emission spectra of CDs were attributed to differences in particle size and nitrogen content. Yuan et al. obtained full-color CDs, spanning blue to red light, by simply changing the reaction time of citric acid and diaminonaphthalene in an ethanol solvent [16]. Their findings indicated that the progressively increasing size of CDs corresponded with the red-shifted fluorescence wavelength, clearly demonstrating the bandgap transitions in CDs. Wang et al. achieved a series of CDs using a scalable acid reagent engineering strategy [17]. They observed that the introduction of electron-withdrawing groups on the surface of CDs, derived from acidic reagents, led to an increased redshift in fluorescence emission and larger particle sizes, thereby elucidating the quantum size effect. Dai et al. developed a pH-controlled method to synthesize multicolor CDs with vibrant violet, green, and orange fluorescence through the hydrothermal treatment of 2,3-diaminopyridine in alkaline, neutral, and acidic aqueous solution, respectively [22]. The redshift observed in fluorescence emissions of these CDs can be attributed to the increased size of the conjugated sp^2^ domain and the higher amount of graphitic nitrogen. These pioneering works have promoted the synthesis of multicolor CDs and the study of the fluorescence mechanism. However, the development of CDs with multicolor emissions is still in the early stage. Therefore, devising a straightforward and effective synthetic strategy for fabricating CDs with tunable multicolor emissions and thoroughly exploring their fluorescence mechanisms remains a significant challenge.

Apart from contributing to the research of fluorescence mechanism, multicolor emission CDs have received increasing attention for LEDs. LEDs that utilize phosphors offer significant potential for applications in liquid crystal displays and multicolor displays due to their advantages in energy conservation, rapid response speed, long lifespan, and high energy efficiency [23]. A wide range of fluorescent materials have been employed as phosphors in LEDs, including semiconductor quantum dots [24,25], rare earth-based nanoparticles [26,27], organic fluorescent dyes [28], and so on. Whereas, high cost, high toxicity, susceptibility to photobleaching, and the challenging synthesis of these materials seriously limit their practical application in LEDs. As a novel type of fluorescent nanomaterial, CDs have emerged as promising alternatives to traditional LEDs phosphors thanks to their excellent fluorescence tunability, low toxicity, high thermal/photo stability, and good environmental compatibility [29,30]. Especially, the synthesis of multicolor CDs provides the potential for fabricating colorful LEDs and white LEDs [31,32,33].

**Scheme 1:**
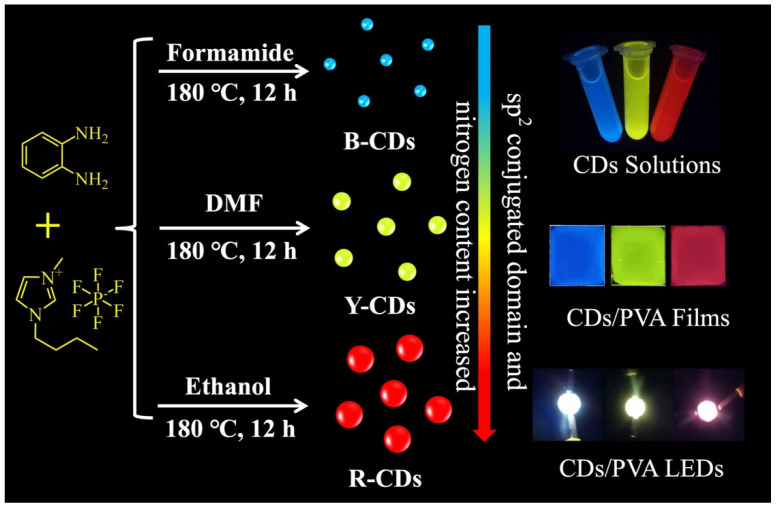
Schematic illustration of the preparation and application of multicolor carbon dots.

Based on our previous work [2], we found that blue- and yellow-emitting CDs could be synthesized by simply adjusting the reaction solvents in solvothermal route. In other words, blue-, yellow-, and red-emitting CDs were prepared by heating *ο*PD and ionic liquid BmimPF_6_ in formamide, dimethylformamide (DMF), and absolute ethanol, respectively. A series of detailed characterizations indicated that the differences in fluorescence of CDs, ranging from blue to red, derived from their increased carbon cores with sp^2^-conjugated domains and nitrogen doping content, which can be regulated by controlling the dehydration and carbonization processes in the solvothermal reaction. The carbon core with a larger sp^2^-conjugated domain exhibited lower bandgaps, causing a redshift in the fluorescence emission. Furthermore, the introduction of surface functional groups on CDs can narrow the bandgap, contributing to the redshift in fluorescence emission. Additionally, blue, yellow, and red CDs-based PVA films were obtained by combining the prepared CDs with PVA, and multicolor LEDs were assembled by commercial LED chips, demonstrating significant potential for applications in the field of multicolor LEDs (Scheme 1).

## 2. Results and Discussion

### 2.1. Optical Properties of B-CDs, Y-CDs, and R-CDs

**Figure 1:**
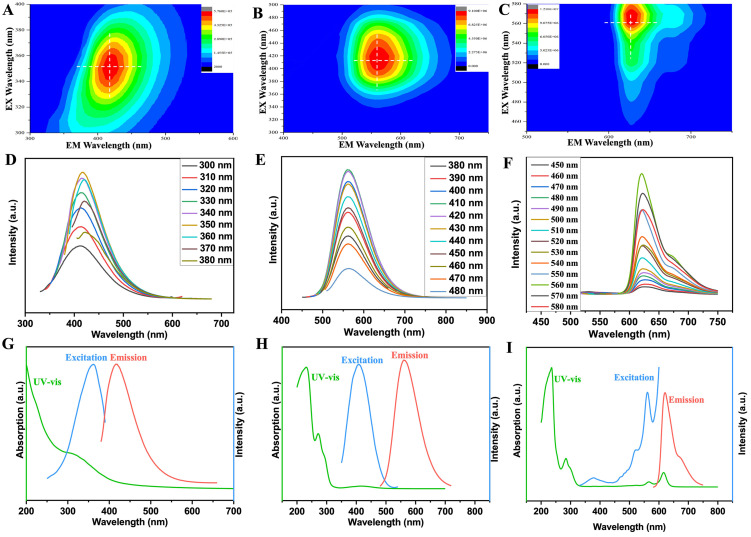
Excitation–emission matrix for (**A**) B-CDs, (**B**) Y-CDs, and (**C**) R-CDs. Fluorescence emission spectra of (**D**) B-CDs, (**E**) Y-CDs, and (**F**) R-CDs under excitation of different wavelength. UV-vis absorption, fluorescence excitation, and emission spectra of (**G**) B-CDs, (**H**) Y-CDs, and (**I**) R-CDs.

Firstly, the optical properties of multicolor CDs were measured by UV–vis absorption spectroscopy, fluorescence excitation, and emission spectra. According to the excitation–emission matrix in Figure 1A–C, the emission centers were located in the blue, yellow and red region, respectively. R-CDs, B-CDs, and Y-CDs all exhibited excitation-independent fluorescence emission properties within different excitation wavelengths (Figure 1D–F). The maximum fluorescence emission peaks were centered at 415 nm for B-CDs, 560 nm for Y-CDs, and 620 nm for R-CDs, with optimal excitation wavelengths of 350, 410, and 560 nm, respectively. To further explore the optical properties of the synthesized CDs, the International Commission on Illumination (CIE) color coordinates (x, y) were calculated using CIE 1931 based on the fluorescence emission spectra of each individual CD. As shown in Figure S1, the prepared colorful CDs occupied distinct regions of the CIE 1931 color space, corresponding to blue, yellow, and red. In the ethanol solution, these CDs exhibited bright fluorescence ranging from blue to red under UV light (excited at 365 nm), which was readily visible to the naked eye (Figure S1, inset). The absolute QYs of B-CDs, Y-CDs, and R-CDs in the ethanol solution were calculated to be 45.3%, 38.7%, and 27.5%, respectively, by using an integrating sphere under their optimal excitation wavelengths. Additionally, time-resolved fluorescence measurement was conducted for each sample. As illustrated in Figure S2 and Table S1, the fluorescence decay curve was analyzed using a single exponential or double exponential formula, giving lifetimes of 4.15, 2.12, and 1.76 ns for B-CDs, Y-CDs, and R-CDs, respectively. The observed decrease in the fluorescence lifetime with the redshift of the emission wavelength suggested that the larger sp^2^ domain in the CDs led to a faster fluorescence decay [34]. The UV-vis absorption spectra of three CDs depicted similar absorption peaks in the UV region, while significant differences in the lower-energy region were observed (Figure 1G–I). In the UV region, the absorption peak under 300 nm corresponded to the π–π* transitions of C=C and C=N bonds in carbon cores [35]. However, in the lower-energy region, distinct absorption features were noted: B-CDs exhibited a broad absorption band around 340 nm, Y-CDs displayed a significant absorption band spanning from 350 to 450 nm, and R-CDs showed a prominent absorption band extending from 500 to 700 nm. These variations indicated that the CDs process different surface states. The absorption bands in the lower-energy region were related to the n–π* transitions of C=O and C=N bonds on the surface of CDs, which contributed to the reduction in the bandgaps of these fluorescent materials [22]. Notably, the optimal excitation spectra of the CDs were consistent with the relevant lower-energy absorption bands, as evidenced by their overlapping wavelength regions, revealing the band-edge emission properties of CDs [17]. Meantime, the red-shifted fluorescence from blue to red was strongly associated with the redshift of excitonic absorption bands, which suggested an increase in the extent of sp^2^-conjugated domains within the progressively larger particles [36]. In addition, the stability of CDs under continuous UV irradiation and in a high-concentration salt solution was studied (Figures S3 and S4). The fluorescence intensity exhibited only a slight decrease after 30 min of continuous UV illumination, and remained unchanged in a high-concentration salt solution, indicating the excellent optical stability of CDs.

### 2.2. Morphologies, Structures, and Components of CD

**Figure 2:**
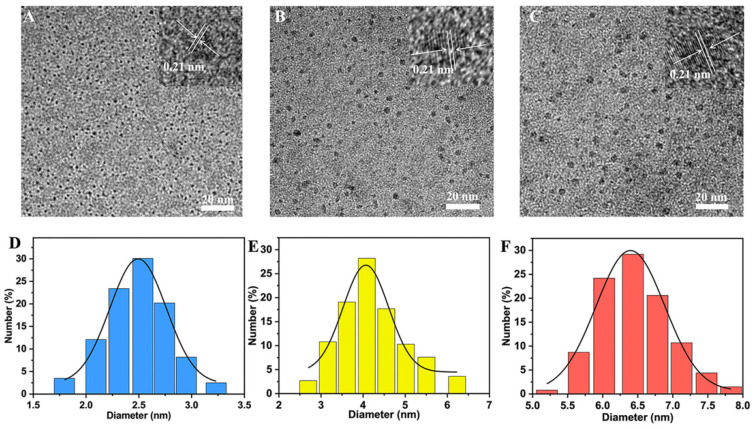
TEM and HRTEM images (inset) of (**A**) B-CDs, (**B**) Y-CDs, and (**C**) R-CDs. Diameter distribution of (**D**) B-CDs, (**E**) Y-CDs, and (**F**) R-CDs.

Subsequently, the morphologies and particle sizes of the as-synthesized CDs were analyzed using TEM. The images revealed that all the CDs were spheroidal and uniformly dispersed (Figure 2A–C). High-resolution TEM images further illustrated that the high crystallinity structure of CDs with identical well-resolved lattice fringes (insets in Figure 2A–C). The crystal plane spacing of 0.21 nm corresponded to the (100) lattice distance of the graphite carbon [37,38]. As depicted in Figure 2D–F, the average diameters of B-CDs, Y-CDs, and R-CDs were estimated to be 2.5, 4.0, and 6.5 nm, respectively. Notably, the gradual increase in the size of CDs coincided with a shift of the emission spectra towards longer wavelengths, revealing that the quantum confinement effect may be responsible for the red-shifted fluorescence wavelength [36].

**Figure 3:**
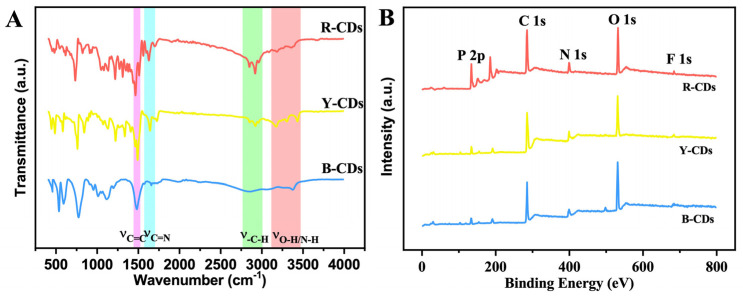
(**A**) FTIR and (**B**) XPS of B-CDs, Y-CDs, and R-CDs.

To gain insight into the functional groups and surface composition of CDs, FTIR and XPS spectra were further investigated. The FTIR spectra indicated similar chemical structures on the surface of the as-prepared CDs (Figure 3A). The broad absorption bands observed at 3100 to 3500 cm^−1^ and 2800 to 3000 cm^−1^ corresponded to the stretching vibrations of O–H/N–H and C–H, respectively. The stretching vibration bands at 1500 cm^−1^ (C=C) and 1630 cm^−1^ (C=N) increased progressively with the redshift of emission wavelengths, suggesting that R-CDs contained a higher degree of sp^2^-conjugated domain and nitrogen doping compared to Y-CDs and B-CDs [5]. The XPS survey spectra indicated that B-CDs, Y-CDs, and R-CDs primarily contained C, N, O, P, and F elements, with binding energies located at 285, 400, 532, 133 and 686 eV, respectively (Figure 3B). The atomic percentages of these elements were detailed in Table S2. As the emission wavelength shifts to longer wavelengths, the proportion of O atoms decreases from 30.94% to 23.26%. Conversely, the N content increases from 8.01% to 9.91%, indicating a significant enhancement in nitrogen doping of R-CDs after solvothermal reaction. This finding aligned well with the FTIR results.

**Figure 4:**
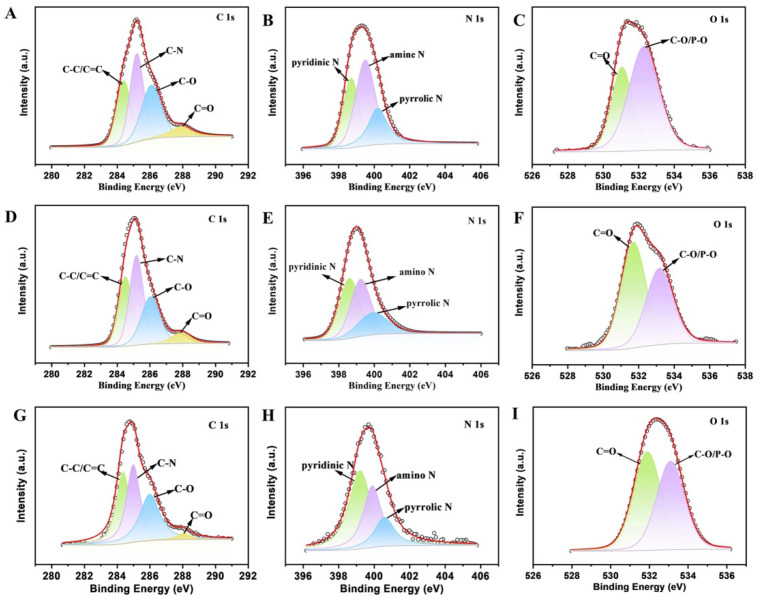
High-resolution XPS spectra of B-CDs (**A**–**C**), Y-CDs (**D**–**F**), and R-CDs (**G**–**I**) for C 1s, N 1s, and O 1s.

In the high-solution XPS spectra, all of the C 1s band of CDs can be divided into four distinct energy peaks, representing C–C/C=C at 284.4 eV, C–N at 285.1 eV, C–O at 286.1 eV, and C=O at 287.9 eV (Figure 4A,D,G) [22]. All the N 1s band of CDs displayed three peaks at 398.7, 399.5, and 400.2 eV, corresponding to pyridinic N, amino N, and pyrrolic N, respectively [15,18] (Figure 4B,E,H). The O 1s band contained two peaks assigned to C=O (531.5 eV) and C–O/P–O (533.1 eV), respectively [23] (Figure 4C,F,I). The proportion of C–C/C=C in the C 1s spectrum gradually increased from 27.0 in B-CDs to 31.2% in R-CDs (Table 1), reflecting an increasing degree of sp^2^-conjugated domains [39], which was consistent with the FTIR results. Simultaneously, the content of pyridine N increased significantly from 30.2% in B-CDs to 43.5% in R-CDs, while the content of amino N decreased from 46.1% to 35.1% in B-CDs to R-CDs. Therefore, the FTIR and XPS analysis results indicated that the synthesized colorful CDs possessed abundant oxygen- and nitrogen-containing groups on their carbon core surface after the dehydration reaction, and the increased sp^2^-conjugated domains and enhanced nitrogen contents primarily contributed to the fluorescence emission shift from blue to red.

### 2.3. Possible Formation Mechanism and Emission Mechanism of CDs

In this work, three distinct emitting CDs were synthesized by only changing the solvents. Therefore, a deep understanding of the influence of solvents on morphology and performance of CDs during the reaction is crucial. Previous studies demonstrated that the properties of reaction solvents, including solubility, dehydration capacity, polarity, boiling point, and protic or aprotic characteristics, significantly influenced the dehydration and carbonation of carbon source during the solvothermal process [40]. As we all know, the solvothermal methods typically involve sealing organic solvents of a certain volume into a completely closed reactor, which are heated above their boiling points to increase the autogenous pressure and allow product properties to be modified by adjusting variables such as reaction time, temperature, and solvent type [41]. The precursor ultimately formed CDs with different emission wavelength through a series of processes, including dehydration, condensation, polymerization, and carbonization, under high temperature and pressure conditions [42]. Formamide, DMF, and ethanol have boiling points of 210 °C, 153 °C, and 78 °C, respectively. In a reaction space with constant temperature and time, the low boiling point of ethanol resulted in a higher vapor pressure within the autoclave, which easily improved the degree of dehydration and carbonization process, forming CDs with longer wavelength. Conversely, formamide, with the highest boiling point and lowest reaction pressure, produced CDs with the shortest wavelength due to the lowest extent of dehydration and carbonization. The experiment results agreed well with the previous reports, which demonstrated that a lower boiling point of reaction solvent promotes the formation of carbon cores with sp^2^-conjugated domains leading to longer wavelength emission [35]. These findings were consistent with TEM results, where R-CDs synthesized in ethanol solution exhibited the largest particle size, while B-CDs synthesized in formamide solution had the smallest particle size. Therefore, choosing a solvent with an appropriate boiling point is an effective strategy for synthesizing multicolor CDs in a solvothermal route.

Apart from the significant impact of the solvent boiling point on CD formation, the polarity of the solvent also plays a crucial role in determining the solubility of the precursor, which in turn affects the fluorescence properties of the prepared CDs. Formamide and DMF enhance the solubility and stable dispersion of *o*PD and ionic liquid BmimPF_6_ due to the solvation effects. Specifically, the methyl groups in DMF create steric hindrance that aids in the dispersion of precursors. In contrast, formamide, which act as both a proton donor and receptor, tends to form dimers, thereby reducing its interaction with the precursors. Ethanol, being a protonic polar solvent, has strong hydrogen bonding interactions between solvent molecules, which makes it difficult for the precursors to disperse effectively. Therefore, the solubility of *o*PD and ionic liquid BmimPF_6_ in DMF, formamide, and ethanol decreases as the solvent polarity increases, which caused the emission of the resulting CDs to shift toward longer wavelengths. It is important to note that the formation of CDs was influenced by a combination of factors: the solubility, solvent polarity, and boiling point. Although DMF’s high solubility can promote the formation of smaller carbon cores, its lower boiling point can contribute to the formation of larger carbon cores. As a result, the overall effects of DMF resulted in the production of larger carbon cores compared to those formed using formamide [39]. Therefore, the CDs synthesized with formamide, DMF, and ethanol exhibited a redshift emission. In summary, the possible mechanism for CD formation involves further cross-linking and carbonization of the initial polymer dots generated from the dehydration reactions of the precursors, which ultimately transformed into CDs. The dehydration and carbonization degree strongly depended on the solvent property, which led to variation in the size of sp^2^-conjugated domains and the surface state, thereby affecting the emission characteristics of the resulting CDs. The proposed solvent-controlled strategy for preparing multicolor CDs offers a valuable approach for precisely regulating the morphology and fluorescence properties of CDs by selecting appropriate solvents. Gaining a deeper understanding of how solvents impact the morphology and properties of CDs will enable the full exploitation of their advantages and broaden their potential applications.

Although the fundamental mechanisms behind the fluorescence of CDs remain controversial and even contradictory, two main theories prevail: the carbon core state and the surface state [43]. In our study, the TEM results indicated that the fluorescence redshift of CDs depended heavily on the particle sizes and the extent of sp^2^-conjugated domains. Meanwhile, the FTIR and XPS results revealed that as fluorescence emission shifted from blue to red, there was a corresponding increase in sp^2^-conjugated domains and nitrogen content. Therefore, based on the detailed characterizations and relevant studies, we believed that the emission wavelength of CDs was primarily governed by the size of sp^2^-conjugated domains and the enhanced nitrogen contents, which was ascribed to the surface state. Larger sp^2^-conjugated domains and higher nitrogen content led to a narrower energy gap, resulting in a redshift of the fluorescence emission.

To support the aforementioned results, bandgap energies (*E*_g_) of the as-prepared CDs were calculated using the equation *E*_g_ = 1240/λ_edge_, where λ_edge_ represents the onset value of the first excitonic absorption peak in the direction of longer wavelength [16]. In the sequence of B-CDs, Y-CDs, and R-CDs, the calculated bandgap energies decreased progressively from 3.10 to 2.48 to 1.89 eV, as the particle size increased from 2.5 to 4.0 to 6.5 nm, further demonstrating the significant size-dependent property of bandgap energies. Meanwhile, cyclic voltammetry (CV) measurements were performed to investigate the frontier molecular orbital (FMO) energy levels of three CDs. The energy levels of highest occupied molecular orbital (HOMO) and lowest unoccupied molecular orbital (LUMO) for each CDs were determined based on the onset values of the oxidation and reduction peaks. Initially, it was crucial to identify the positions of these oxidation and reduction peaks for the CDs. Take R-CDs as an example: a CV test was conducted by gradually adding R-CDs to a 0.1 M Bu_4_NPF_6_/acetonitrile solution, which had been deoxygenated using nitrogen. As depicted in Figure S5A,B, the oxidation peak current around 0.5 V and the reduction peaks current at about −1.7 V were gradually enhanced with the concentration of R-CDs increasement. This observation confirmed that the two positions correspond to the oxidation and reduction peaks of R-CDs. The onset potentials for the oxidation (*E*_ox_) and reduction (*E*_red_) of R-CDs were noticed at 0.38 and −1.52 V, respectively (Figure S5C,D). Consequently, the energy levels of the HOMO and LUMO for R-CDs were calculated to be −4.78 and −2.88 eV, respectively, using the empirical formula *E*_HOMO_ = −e(*E*_ox_ + 4.4) and *E*_LUMO_ = −e(*E*_red_ + 4.4) [44]. The energy gap (*E*_g_) was computed to be 1.90 eV according to the equation *E*_g_ = *E*_LUMO_ − *E*_HOMO_, in line with the above determined value of 1.89 eV. Similarly, the *E*_HOMO_ and *E*_LUMO_ of Y-CDs were calculated to be −5.62 and −3.10 eV based on their *E*_ox_ of 1.22 V and *E*_red_ of −1.30 V, respectively (Figure S6). Concurrently, *E*_g_ was calculated to be 2.52 eV. However, for B-CDs, only an oxidation peak was observed at approximately1.3 V, with no distinct reduction peak observed within the potential window range of −2.1 to 0 V (Figure S7A,B). The onset oxidation potential of B-CDs was noticed at 1.10 V (Figure S7C), and the *E*_HOMO_ was determined to be −5.46 eV. The LUMO energy could be calculated according to *E*_LUMO_ = *E*_g_ + *E*_HOMO_, with *E*_g_ estimated to be 3.10 eV. Thus, the LUMO energy of B-CDs was determined to be −2.36 eV. The larger size of sp^2^-conjugated domains in CDs reduces the HOMO-LUMO energy gap, leading to a shift in the fluorescence emission from blue to red. Additionally, strong N-doping introduces deep levels within the bandgap, lowing the energy of electronic transitions. Therefore, based on the fluorescence properties, TEM, FTIR, XPS results, and molecular orbital energy levels calculations, it can be concluded that the fluorescence characteristics of CDs were primarily influenced by carbon cores with sp^2^-conjugated domains and the increased nitrogen contents. Larger carbon cores resulted in lower bandgaps, causing a redshift in fluorescence emission. It is worth noting that the size of the sp^2^-conjugated domain, rather than particle size, is responsible for the quantum confinement effect. Furthermore, the introduction of surface functional groups on CDs further narrowed the bandgap, contributing to the redshift in emission wavelength.

### 2.4. Application of CDs/PVA Composite Films and Multicolor LEDs

**Figure 5:**
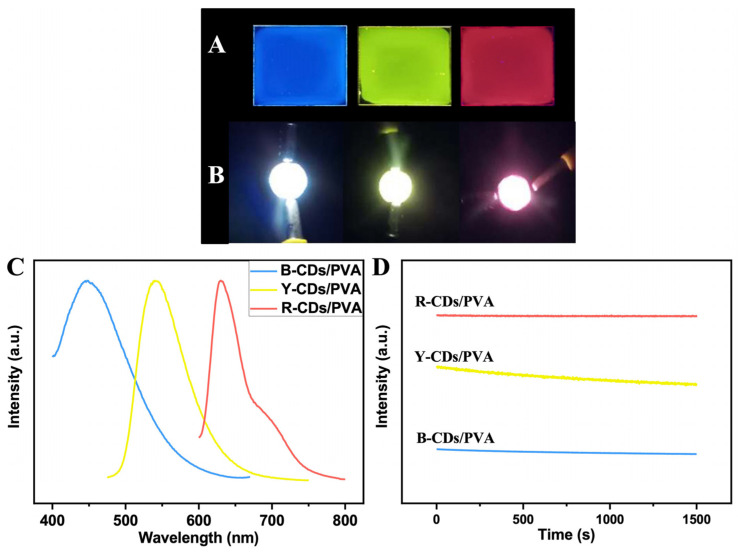
Photograph of the three kinds (**A**) CDs/PVA films under UV light irradiation and (**B**) LEDs operating under the voltage of 4.5 V. (**C**) Fluorescence spectra of the three kinds CDs/PVA films. (**D**) Changes in fluorescence intensity of CDs/PVA LEDs with continuous irradiation.

Although many CDs with high quantum yields in aqueous solution had been developed, most reported CDs exhibited weak or negligible fluorescence in the solid state due to the aggregation-induced quenching, which strictly restricted the development of CDs in LEDs assemblies [45,46]. To overcome this issue, several research groups had fabricated CDs/polymer composites using water-soluble polymers such as PVA, poly(vinyl pyrrolidone) (PVP), poly(acrylic acid) (PAA), and starch to maintain their fluorescence properties [47,48]. In this work, we dispersed CDs in PVA, which contained a large number of hydroxyl groups and a certain length of polymer chain, to prevent the aggregation-induced fluorescence quenching. As shown in Figure 5A, the maxima emission peaks of the CDs/PVA films were observed at 440, 550, and 630 nm, almost the same with the peaks of CDs in an aqueous state. Under a UV light of 365 nm, the films emitted bright blue, yellow, and red fluorescence visible to the naked eyes (Figure 5C). Additionally, the fluorescence intensities of these CDs/PVA composite films remained at almost 85% after 25 min of continuous irradiation, demonstrating excellent photo-stabilities (Figure 5D). Therefore, our multicolor CDs/PVA composite films show great promise as candidates for lighting devices. We further demonstrated this potential by fabricating colorful LEDs using CDs/PVA composites coated on the commercial UV chips (365 nm). The resulting LEDs, as shown in Figure 5B, highlight the application potential of these CDs in the field of multicolor LEDs.

## 3. Materials and Methods

### 3.1. Reagents and Instruments

All reagents and instruments utilized in this work are provided in the Supporting Information.

### 3.2. Preparation of B-CDs, Y-CDs, and R-CDs

Tunable fluorescent CDs were synthesized through a facile one-pot solvothermal method, utilizing *ο*PD and ionic liquid BmimPF_6_ as precursors. The resulting blue-, yellow-, and red-emitting CDs were labeled as B-CDs, Y-CDs, and R-CDs, respectively. The multicolor CDs were synthesized according to our previous study with minor modifications [2]. In brief, *ο*PD (0.24 g) and ionic liquid BmimPF_6_ (1.0 g) were dissolved in formamide, DMF, and ethanol, respectively. Each solution was then transferred into a Teflon-lined stainless steel autoclave and heated at 180 °C for 12 h. After naturally cooling to room temperature, the crude products were purified using different approaches.

The detailed purification process of the prepared CDs is listed in the Supporting Information.

The materials and methods should be described with sufficient details to allow others to replicate and build on the published results.

### 3.3. Preparation of the CDs-Based PVA Films

To fabricate blue, yellow, and red light-emitting films, 2 mL of an ethanol solution containing B-CDs, Y-CDs, or R-CDs (2.0 mg/mL) was mixed with 4 mL of a PVA aqueous solution (10 wt%) under stirring to form the CDs/PVA solutions, respectively. The CDs/PVA solutions were then dropped into clean glass sheets and dried for overnight under ambient conditions. The resulting films were carefully peeled off from the glass substrates and stored in a desiccator for further use.

### 3.4. Fabrication of CDs/PVA-Based LEDs

UV-LED chips with the peak emission wavelength centered at 365 nm were employed for fabricating full-color LEDs. The CDs/PVA solutions were carefully added dropwise onto the top of the chips. Following natural air-drying, the CDs/PVA-based LEDs were successfully assembled.

## 4. Conclusions

In summary, blue, yellow, and red fluorescent CDs were synthesized using *o*PD and ionic liquid BmimPF_6_ as precursors, with the only variable being the solvent type (formamide, DMF, and ethanol). All the synthesized CDs exhibited an excitation-independent fluorescence emission property, and their absolute quantum yields were 45.3%, 38.7%, and 27.5%, respectively. The detailed characterizations proved that the multicolor emission of CDs were dependent on the size of the sp^2^-conjugated domains and the degree of nitrogen doping. Moreover, we studied the influence of solvent properties, such as the boiling point and solubility, on the morphology and performance of CDs and their molecular orbital energy levels. Solvents with a lower boiling point and a lower solubility of precursors resulted in a higher degree of dehydration and carbonization processes, thereby increasing the carbon cores with sp^2^-conjugated domains and nitrogen doping, and further shrinking the bandgap energies between the HOMO and LUMO levels, causing a significant redshift in the fluorescence emission from blue to red. The underlying fluorescence mechanisms of the prepared multicolor CDs was attributed to the surface state. By dispersing the as-synthesized CDs into a PVA solution, we successfully fabricated CDs-based PVA films with tunable colors and colorful LEDs devices, effectively expanding the application potential of CDs.

## Figures and Tables

**Table 1:** XPS data analysis of the C 1s and N 1s spectra of B-CDs, Y-CDs, and R-CDs.

		B-CDs	Y-CDs	R-CDs
C 1s	C–C/C=C (%)	27.0	29.3	31.2
	C–N (%)	34.5	37.5	31.8
	C–O (%)	30.6	27.0	32.3
	C=O (%)	7.9	6.2	4.7
N 1s	pyridinic N (%)	30.2	40.2	43.5
	amino N (%)	46.1	37.8	35.1
	pyrrolic N (%)	23.7	22.0	21.4

## Data Availability

Data are contained within the article and Supplementary Materials; further inquiries can be directed to the corresponding author.

## Supplementary Materials

The following supporting information can be downloaded at https://www.mdpi.com/article/10.3390/molecules29194552/s1, Experimental details (including reagents and materials; instruments; purification of B-CDs, Y-CDs and R-CDs; quantum yield measurements; cyclic voltammetry measurement); Figure S1: Calculate CIE coordinates from the fluorescence spectra of B-CDs, Y-CDs and R-CDs; Figure S2: Time-resolved fluorescence spectra of B-CDs, Y-CDs and R-CDs; Figure S3: Changes in fluorescence intensity of B-CDs, Y-CDs and R-CDs with continuous irradiation for 30 min; Figure S4: Effect of ionic strength on fluorescence intensity of B-CDs, Y-CDs and R-CDs; Figure S5: Cyclic voltammogram of R-CDs in 0.1 M Bu_4_NPF_6_/acetonitrile solution at 100 mV s^−1^; Figure S6: Cyclic voltammogram of Y-CDs in 0.1 M Bu_4_NPF_6_/acetonitrile solution at 100 mV s^−1^; Figure S7: Cyclic voltammogram of B-CDs in 0.1 M Bu_4_NPF_6_/acetonitrile solution at 100 mV s^−1^; Table S1: Fitted parameters of time-resolved fluorescence decay curves of B-CDs, Y-CDs and R-CDs; Table S2: Relative contents of C, N, O, P and F atoms of B-CDs, Y-CDs and R-CDs (determined by XPS). References [49,50,51,52] are citation in Supplementary Materials.
