# Supplementary material for: Solvent-Controlled Strategy for Color-Tunable Fluorescence Carbon Dots and Their Application in Light-Emitting Diodes
Source: Molecules. 2024 Sep 25;29(19):4552. doi: 10.3390/molecules29194552 (PMC11477868; doi:10.3390/molecules29194552)
Supplement: Supplementary file 1 [file molecules-29-04552-s001.zip › molecules-3204018-supplementary.pdf]

Supporting Information

Solvent-Controlled Strategy for Color-Tunable Fluorescence Carbon Dots and Their Application in Light-Emitting Diodes

Yuhua Zhang[1,2], Hong Zhao[2]*

[1]*School of Pharmacy, Shandong Second Medical University, Weifang 261053, China*

[2]*School of Chemical Sciences, University of Chinese Academy of Sciences, Beijing 100049, China*

* Corresponding author:

E-mail: hongzhao@ucas.ac.cn (H. Zhao), Tel.: +86-10-88256093.

Table of Contents

1. Experimental details.

2. Calculate CIE coordinates from the fluorescence spectra of B-CDs, Y-CDs and R-CDs. (Figure S1)

3. Time-resolved fluorescence spectra of B-CDs, Y-CDs and R-CDs. (Figure S2)

4. Changes in fluorescence intensity of B-CDs, Y-CDs and R-CDs with continuous irradiation for 30 min. (Figure S3)

5. Effect of ionic strength on fluorescence intensity of B-CDs, Y-CDs and R-CDs. (Figure S4)

6. Cyclic voltammogram of R-CDs in 0.1 M Bu_4NPF_6/acetonitrile solution at 100 mV s^{-1}. (Figure S5)

7. Cyclic voltammogram of Y-CDs in 0.1 M Bu_4NPF_6/acetonitrile solution at 100 mV s^{-1}. (Figure S6)

8. Cyclic voltammogram of B-CDs in 0.1 M Bu_4NPF_6/acetonitrile solution at 100 mV s^{-1}. (Figure S7)

9. Fitted parameters of time-resolved fluorescence decay curves of B-CDs, Y-CDs and R-CDs. (Table S1)

10. Relative contents of C, N, O, P and F atoms of B-CDs, Y-CDs and R-CDs (determined by XPS). (Table S2)

Experimental details

Reagents and Materials

Ionic liquids $BmimPF_6$ (99%), BmimDCA (99%), $BmimBF_4$ (99%), BmimCl (99%) were purchased from Linzhou Keneng Material Technology Co., Ltd. *o*PD (99%), DMF, and methylene chloride were obtained from Shanghai Macklin Biochemical Technology Co., Ltd. Ethanol and methanol were supplied by Beijing Chemical Reagent Co., Ltd. Formamide and PVA were obtained from Energy Chemical. Acetone and acetonitrile (chromatographically pure) were provided by Sinopharm Chemical Reagent Beijing Co., Ltd. Tetrabutylammonium hexafluorophosphate (Bu_4NPF_6) were acquired by Sigma-Aldrich. All reagents were used as received without further purification unless otherwise specified. Milli-Q ultrapure water with a resistivity of 18.2 MΩ cm was used for all experiments.

Instruments

Transmission electron microscopy (TEM) was obtained from a Philips EM-400. Fourier transform infrared (FTIR) spectroscopy was recognized with a BRUKE Vertex 70 FT-IR spectrophotometer. X-ray photoelectron spectra (XPS) were performed on Thermo Scientific Escalab 250Xi. The electrochemical measurements were monitored by a CHI 660D electrochemical workstation (Chenhua, Shanghai). UV–vis spectra were recorded on a UV-2550 spectrophotometer. Fluorescence lifetime and QY measurements were carried out on a FLS980 fluorescence spectrometer. Fluorescence spectra were taken on a FluoroMax-4P fluorescence spectrophotometer.

Purification of B-CDs, Y-CDs and R-CDs

For B-CDs, the purified approach was according to the literature [1]. At first, the resulting CDs solution was added into 100 mL of acetone solution and placed in the refrigerator (−20°C) overnight. The precipitation was then washed with acetone and 10% methanol/acetone three times, respectively (25 mL solvent being used for each washing step). Subsequently, the residue was redispersed in methanol and filtered through a 0.22 μm membrane to remove large particles. Finally, the purified B-CDs were obtained by evaporating off methanol and further drying in a vacuum oven.

For Y-CDs and R-CDs, the corresponding crude products was purified with a silica column chromatography using mixtures of methylene chloride and methanol as eluents. After removing the solvents and the final products were dried in a vacuum oven.

Quantum yield (QY) measurements

The absolute quantum yield of the CDs was measured by using an integrating sphere attached to an FLS 980 spectrofluorometer. For instance, the R-CDs ethanol solution, which was diluted to an absorption intensity below 0.1 at excitation wavelength of 560 nm, was added into a 1 cm fluorescence cuvette and placed in the integrating sphere and then excited with 560 nm monochromatic light. Subsequently, the fluorescence spectra were recorded in the ranges of 530-850 and 550-570 nm, respectively. Meantime, the same fluorescence spectra for ethanol were also measured under the same conditions. Finally, the QY was calculated using fluorescence software based on

the fluorescence spectra of both the sample and pure ethanol. Each experiment was conducted by three times in parallel to calculate the average QY. The fluorescence spectra of B-CDs and Y-CDs were recorded on the same way.

Cyclic voltammetry (CV) measurement

The CV measurement was performed using a standard three-electrode system using the CHI 660D electrochemical workstation (Shanghai Chenhua). Glass-carbon electrode was used as the working electrode, Ag/AgCl electrode as the reference electrode, platinum electrode as the counter electrode, and the acetonitrile solvent of tetrabutylammonium hexafluorophosphate (Bu_4NPF_6/acetonitrile solution, 0.1 M) as the electrolyte solution. Before the electrochemical measurement, the electrolyte solution was de-oxygenated with nitrogen, the tetrabutylammonium hexafluorophosphate was used after recrystallization and the acetonitrile solvent was chromatographically pure.

Prior to electrochemical testing, glass-carbon electrode was first sanded on metallographic sandpaper and then polished to a mirror on suede coated with 0.3 and 0.05 μm alumina suspension, respectively, followed by ultrasonic cleaning in ethanol and deionized water, blow-dried with high-purity nitrogen, and tested for CV in potassium ferricyanide solution to ensure a smooth surface. Then CV measurement of R-CDs was tested the acetonitrile solution of tetrabutylammonium hexafluorophosphate (0.1 M). The electrochemical testing of B-CDs and Y-CDs were recorded on the same way.

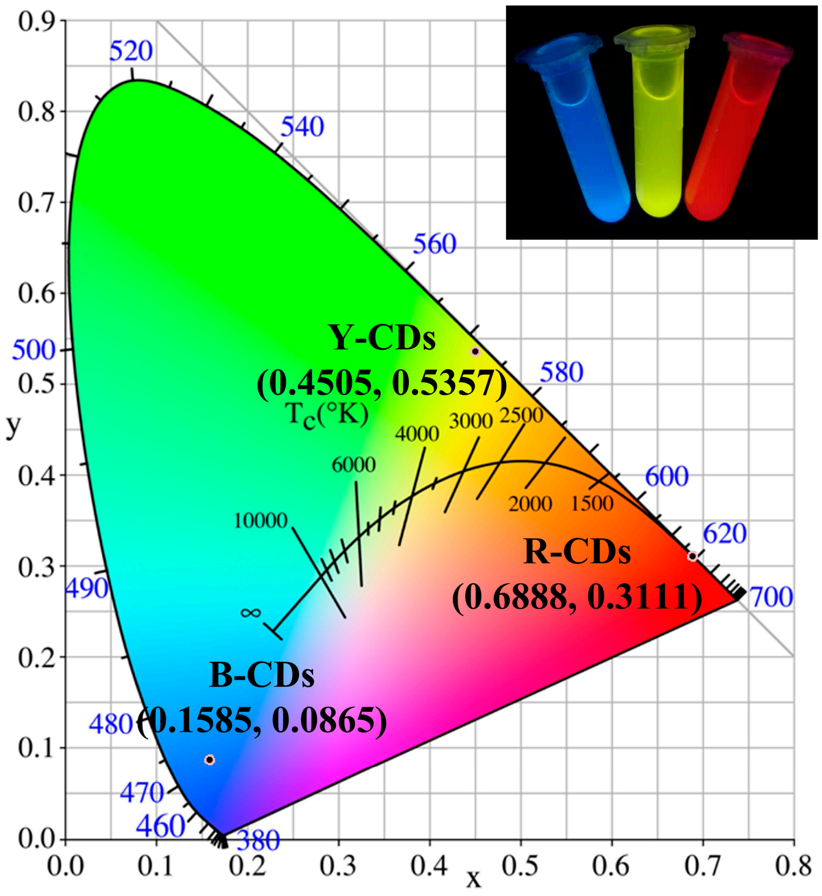

Figure S1. Calculate CIE coordinates from the fluorescence spectra of B-CDs, Y-CDs and R-CDs (inset: photograph of the three kinds CDs under UV light irradiation).

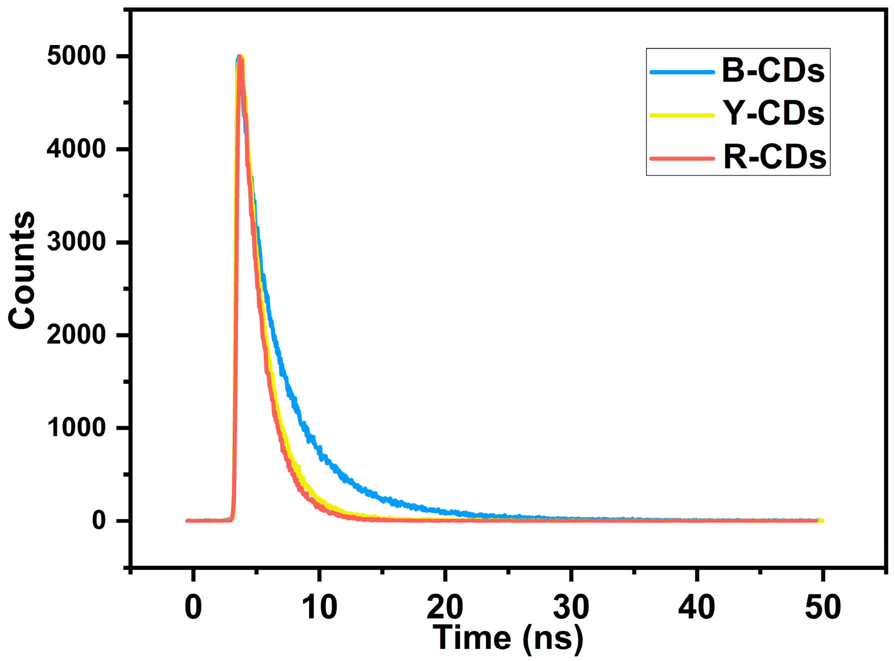

Figure S2. Time-resolved fluorescence spectra of B-CDs, Y-CDs and R-CDs.

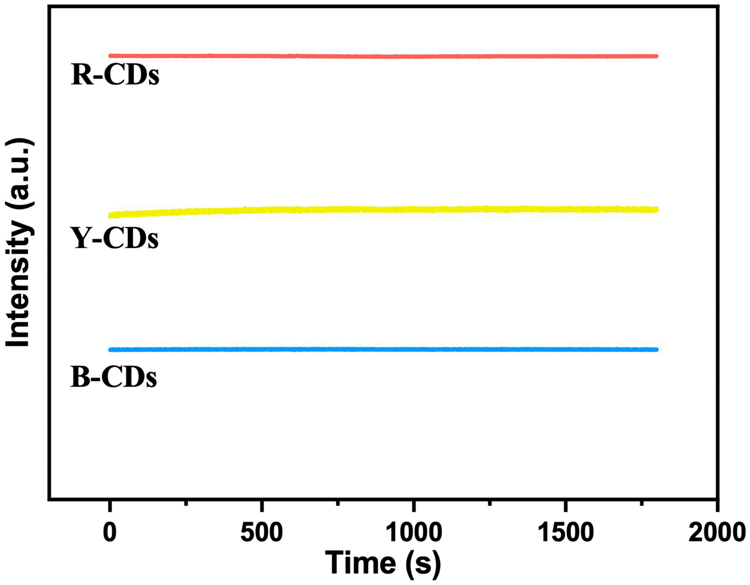

Figure S3. Changes in fluorescence intensity of B-CDs, Y-CDs and R-CDs with continuous irradiation for 30 min.

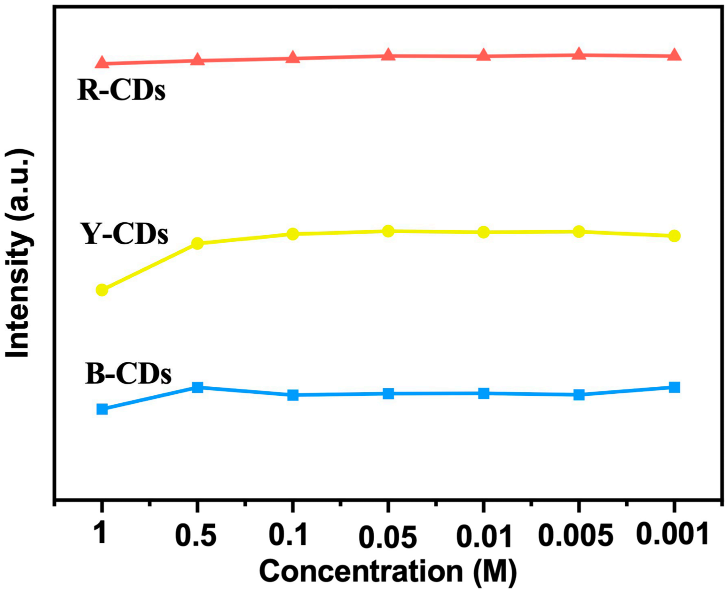

Figure S4. Effect of ionic strength on fluorescence intensity of B-CDs, Y-CDs and R-CDs. $\lambda_{ex}/\lambda_{em}$ = 350 nm/415 nm, 410 nm/560 nm, and 560 nm/620 nm, respectively.

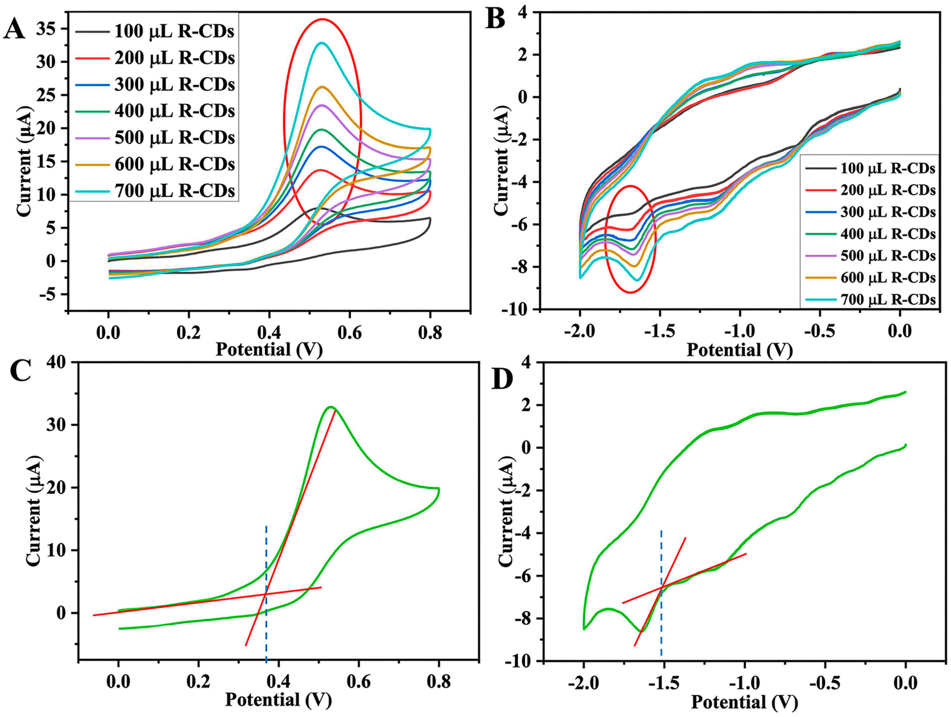

Figure S5. (A) Cyclic voltammogram of R-CDs (different concentrations) in 0.1 M Bu_4NPF_6/acetonitrile solution at 100 mV s^{-1} with the potential window from 0-0.8 V. (B) Cyclic voltammogram of R-CDs (different concentrations) in 0.1 M Bu_4NPF_6/acetonitrile solution at 100 mV s^{-1} with the potential window from -2.0-0 V. (C) Cyclic voltammogram of R-CDs (700 μL of 0.1 M) in 0.1 M Bu_4NPF_6/acetonitrile solution at 100 mV s^{-1} with the potential window from 0-0.8 V. (D) Cyclic voltammogram of R-CDs (700 μL of 0.1 M) in 0.1 M Bu_4NPF_6/acetonitrile solution at 100 mV s^{-1} with the potential window from -2.0-0 V.

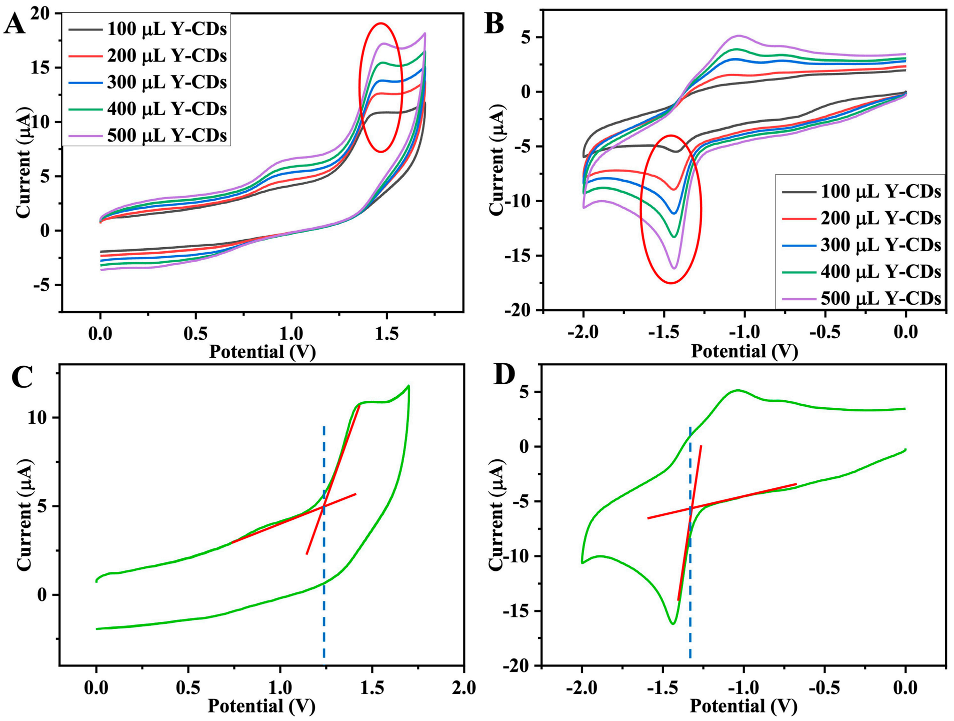

Figure S6. (A) Cyclic voltammogram of Y-CDs (different concentrations) in 0.1 M Bu_4NPF_6/acetonitrile solution at 100 mV s^{-1} with the potential window from 0-1.75 V. (B) Cyclic voltammogram of Y-CDs (different concentrations) in 0.1 M Bu_4NPF_6/acetonitrile solution at 100 mV s^{-1} with the potential window from -2.0-0 V. (C) Cyclic voltammogram of Y-CDs (100 μL of 0.1 M) in 0.1 M Bu_4NPF_6/acetonitrile solution at 100 mV s^{-1} with the potential window from 0-0.8 V. (D) Cyclic voltammogram of Y-CDs (100 μL of 0.1 M) in 0.1 M Bu_4NPF_6/acetonitrile solution at 100 mV s^{-1} with the potential window from -2.0-0 V.

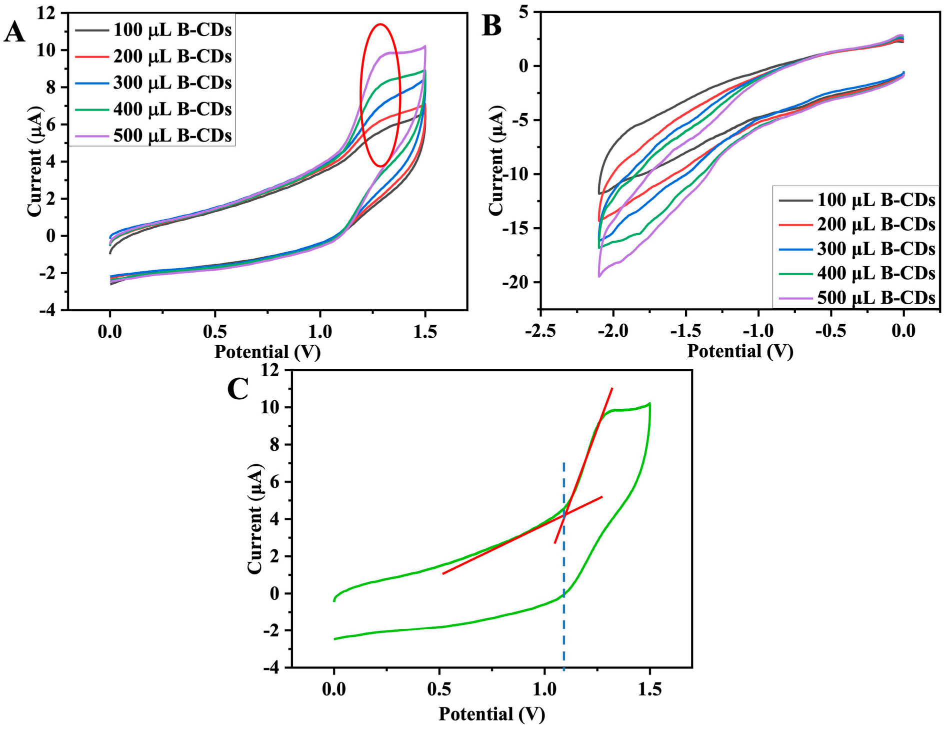

Figure S7. (A) Cyclic voltammogram of B-CDs (different concentrations) in 0.1 M Bu_4NPF_6/acetonitrile solution at 100 mV s^{-1} with the potential window from 0-1.5 V. (B) Cyclic voltammogram of B-CDs (different concentrations) in 0.1 M Bu_4NPF_6/acetonitrile solution at 100 mV s^{-1} with the potential window from -2.0-0 V. (C) Cyclic voltammogram of B-CDs (500 μL of 0.1 M) in 0.1 M Bu_4NPF_6/acetonitrile solution at 100 mV s^{-1} with the potential window from 0-1.5 V.

Table S1. Fitted parameters of time-resolved fluorescence decay curves of B-CDs, Y-CDs and R-CDs.

Samples	B-CDs	Y-CDs	R-CDs
λ_{ex} (nm)	340	410	560
λ_{ex} (nm)	415	560	620
τ_1 (ns)	2.38	1.91	1.76
A_1(%)	49.80	96.31	100
τ_1 (ns)	5.91	7.63	-
A_2(%)	50.20	3.69	-
τ_{avg} (ns)	4.15	2.12	1.76

Fluorescence lifetime measurements was carried out on a FLS980 fluorescence spectrometer. The fluorescence decay curve of R-CDs was fitted by a mono-exponential decay with lifetime of 1.76 ns. The time-resolved fluorescence decay curves of B-CDs and Y-CDs can be fitted by a double exponential formula:

$$R(t) = A_1 \exp\left(-t/\tau_1\right) + A_2 \exp\left(-t/\tau_2\right)$$

where *R(t)* represents the relative intensity of fluorescence, τ_1 and τ_2 represent a short-lived component and a long-lived component, A_1 and A_2 are constants, and t is the decay time [2]. The calculated $\tau_1$values for B-CDs and Y-CDs are 2.38 ns and 1.91 ns, respectively, and τ_2 values for B-CDs and Y-CDs are 5.91 ns and 7.63 ns, respectively. The average τ values for B-CDs, Y-CDs and R-CDs are 4.15 ns, 2.12 ns and 1.76 ns, respectively. The above-mentioned parameters are summarized in Table S1. The

existence of τ_1 and τ_2 imply that CDs have two fluorescence centers originating from $\pi-\pi^*$ transitions of the carbon core with conjugated sp^2 domains and $n-\pi^*$ transitions of O- and N- contained functional groups [3-6], which tune together the fluorescence performance of CDs.

Table S2. Relative contents of C, N, O, P and F atoms of B-CDs, Y-CDs and R-CDs (determined by XPS)

Sample	C (%)	N (%)	O (%)	P (%)	F (%)
B-CDs	58.34	8.01	30.94	1.88	0.83
Y-CDs	60.23	8.73	28.35	2.04	0.65
R-CDs	63.49	9.91	23.26	2.87	0.47

Reference

[1] Sun S, Zhang L, Jiang K, Wu AG, Lin HW. Toward high-efficient red emissive carbon dots: Facile preparation, unique properties, and applications as multifunctional theranostic agents. Chem Mater. 2016;28(23):8659-68.

[2] Bai JL, Ma YS, Yuan GJ, Chen X, Mei J, Zhang Land Ren LL. Solvent-controlled and solvent-dependent strategies for the synthesis of multicolor carbon dots for pH sensing and cell imaging. J. Mater. Chem. C, 2019, 7, 9709–9718.

[3] Ding H, Wei JS, Zhang P, Zhou ZY, Gao QY, Xiong HM. Solvent-controlled synthesis of highly luminescent carbon dots with a wide color gamut and narrowed emission peak widths. Small. 2018;14(22):1800612.

[4] Zheng YX, Arkin K, Hao JW, Zhang SY, Guan W, Wang LL, Guo YN and Shang QK. Multicolor carbon dots prepared by single-factor control of graphitization and surface oxidation for high-quality white light-emitting diodes. Adv. Opt. Mater., 2021, 9, 2100688.

[5] Manioudakis J, Victoria F, Thompson CA, Brown L, Movsum M, Lucifero R and Naccache R, Effects of nitrogen-doing on the photophysical properties of carbon dots. J. Mater. Chem. C, 2019, 7, 853–862.

[6] Zhang SS, Yuan L, Liang GZ, Gu AJ, Preparation of multicolor-emissive carbon dots with high quantum yields and their epoxy composites for fluorescence anti-counterfeiting and light-emitting devices. J. Mater. Chem. C, 2022, 10, 8441-8458.
